# How Can Biomedical Journals Help to Tackle Global Poverty?

**DOI:** 10.1371/journal.pmed.0030380

**Published:** 2006-08-29

**Authors:** The PLoS Medicine Editors

## Abstract

Journals have been slow to realize their potential as a tool for reducing poverty and addressing global inequities, say the*PLoS Medicine* editors.

Out of the eight United Nations Millennium Development Goals (http://www.un.org/millenniumgoals), one of the most crucial is the goal of halving the number of people living in extreme poverty—most of whom live in developing countries and who survive on less than US$1 per day—by 2015. The scientific and medical communities have an important role to play in reaching this goal through, for example, tackling the infectious diseases that promote poverty (such as HIV/AIDS, malaria, and intestinal worms), reversing the loss of environmental resources, and disseminating new technologies to developing countries. And scientific and medical journals, the “arbiters of formalized scientific knowledge” (http://ejournal.nbii.org/archives/vol2iss1/editorial.bozuwa.html), are central to this enterprise.

Unfortunately, journals have been slow to realize their potential as a tool for reducing poverty and addressing global inequities. Journal editors and publishers have shown a bias against publishing materials on the diseases of poverty (Lancet 361: 712–713). They have done poorly at featuring the work of researchers from developing countries (BMC Med Ethics 5: 5) and at including such researchers on their editorial boards (BMJ 328: 1229–1232). And, perhaps most shamefully, they have failed to heed the United Nations' repeated calls to provide universal access to the scientific and medical literature (http://www.unmillenniumproject.org/reports/tf_science.htm).

Why have journals shown so little interest in the problems of the developing world? One obvious reason is that most journals make a profit by selling their content to readers (a single article from one of the big publishers typically costs a non-subscriber US$30–US$50 to read; subscriptions for a year are usually many hundreds of US dollars), so to remain profitable these journals are forced to publish materials that will appeal to readers who can pay. Another major source of revenue for many journals is advertising “blockbuster” drugs to doctors in affluent countries (PLoS Med 3: e130), and it is highly unlikely that drug companies would pay for such adverts if the journals had a major focus on the diseases of the poor world. As long as journals rely on such a “reader pays” model and on drug advertising, their hands will always be tied—they will have to prioritize articles that focus on the health problems of the rich world.

Journals have been slow to realize their potential as a tool for poverty reduction.

Another reason is that editors have traditionally taken a very narrow position on the role of biomedical journals. In his opening address at this year's Council of Science Editors annual meeting, Richard Horton, editor of *The Lancet* and current president of the council, said that some editors feel that addressing global challenges is “lofty and over-ambitious and not our concern.” Rejecting such a “restricted view,” Horton argued that editors can make an important contribution to these challenges by identifying gaps, weaknesses, and failures in our scientific knowledge and convening partnerships to address these; being advocates and leaders; helping to create and sustain social movements; and helping to educate and inform scientists and the public.

In order to sensitize journal editors to the problem of tackling extreme poverty, the Council of Science Editors has established the Task Force on Science Journals, Poverty, and Human Development (http://www.councilscienceeditors.org/services/taskforce.cfm), of which *PLoS Medicine* is a member. The task force is advocating that journal editors become more engaged in helping to achieve the Millennium Development Goals, and is drafting a set of principles outlining editors' responsibilities to the developing world. The task force is encouraging editors to make their journal contents more accessible and relevant to those in developing countries, and to ensure greater participation from scientists and health professionals in these countries as authors, peer reviewers, and editorial board members. One important initiative to emerge from the task force is a plan for at least 100 journals to simultaneously publish articles relevant to poverty towards the end of 2007. (Of the PLoS journals, *PLoS Medicine*, *PLoS Biology*, and *PLoS Clinical Trials* have all agreed to take part.)

The task force is also examining ways to foster local research and publishing capacity in low-income countries, for example by helping to launch Author Aid, an initiative to mentor authors from developing countries who are preparing research papers for local or international publication (http://www.jphp.umb.edu/documents/Authoraid.pdf). In a debate in this issue of *PLoS Medicine* on whether local journals still serve a function in the new era of online international journals, David Ofori-Adjei, Editor-in-Chief of the *Ghana Medical Journal*, reminds us that local journals still have a vital part to play in disseminating local knowledge, translating it into policy and practice, and contributing to national development.

The establishment of the Council of Science Editors' task force is a promising sign that journals are finally recognizing the ways in which they can contribute to human, social, and economic development. Another sign is that more than 3,000 scientific and medical journals have joined HINARI, the Health InterNetwork Access to Research Initiative (http://www.who.int/hinari/en). This initiative gives free access to these journals to non-profit institutions in countries with a gross national product (GNP) per capita below $1,000, provided the institution agrees to the rules of the Berne Convention (http://www.wipo.int/treaties/en/ip/berne/summary_berne.html), which restrict how the journal contents can be used. Institutions in countries with a GNP per capita between $1,000 and $3,000 pay a fee of $1,000 per year per institution for access to the HINARI journals.

While HINARI is a welcome initiative, it has several major limitations. First, the GNP cut-off excludes many of the world's most populous nations where the need for current medical and scientific information is especially acute, including Brazil, China, India, and Indonesia. Second, the copyright laws that apply to these 3,000 or so journals prohibit readers from reproducing, sharing, or translating the materials—a particularly severe obstacle in countries in which Internet access is unreliable. Third, only non-profit institutions are eligible—so an individual clinician, researcher, or teacher cannot reap the benefits.

A more radical step that journals could take to address global inequities is for them to back the United Nations' call for open access, which would grant readers worldwide the unlimited right to freely download, distribute, and translate materials and to create derivative works. Developing countries are improving their capacity to harness scientific and technical knowledge to solve local problems themselves, and editors of both local and international journals could support these countries in this process by expanding the world's pool of public domain knowledge. Expanding this “knowledge commons” would give developing countries the scientific and technical information needed to solve fundamental challenges, promote public health, manage the environment, and participate in international trade.

And, as Elizabeth Slade (Senior Editor, Sciencenow) and Pritpal Tamber (Managing Editor, Faculty of 1000 Medicine) argue in their contribution to this month's *PLoS Medicine* debate, open-access journals can be truly international in what they publish since they “need not concern themselves with choosing content that would appeal to wealthy audiences.” Open access thus gives authors in all countries the opportunity to contribute to the global scientific debate, to the knowledge commons, and to setting the science and health agenda.

Championing open access is arguably the most effective way that journals can help to lift people out of the extremes of poverty.

## Abbreviations

GNP: gross national product

